# Applying Telemedicine Technology in Treating Prolactinomas: A Case Report

**DOI:** 10.7759/cureus.9043

**Published:** 2020-07-07

**Authors:** Hiba Khalil, Rayyan Abdelnabi, Ahmed Osman, Waiel A Bashari

**Affiliations:** 1 Radiology, University of Khartoum, Khartoum, SDN; 2 Radiology, Leeds University Hospitals NHS Trust, Leeds, GBR; 3 Institute of Metabolic Science, Cambridge University Hospital, Cambridge, GBR

**Keywords:** prolactinoma, telemedicine, tele-consultation

## Abstract

Despite being considered a relatively new concept, telemedicine has already been associated with improved outcomes and reduced healthcare utilization in the management of several high-risk diseases. However, no reports to date have examined the effectiveness of telemedicine in managing prolactinomas. We report a case of a young male with a macroprolactinoma who was reviewed initially in a face-to-face encounter, however, continued his subsequent management virtually using telemedicine methods with satisfactory clinical outcomes.

## Introduction

Prolactinomas are the most common subtype of pituitary adenomas, affecting females more than males [1]. They may present with symptoms of galctorrhoea, hypogonadism (e.g. amenorrhoea) or pressure effects into nearby structures (e.g. the optic chiasm, causing visual field defects). Alternatively, prolactinomas can be detected incidentally during brain imaging. In addition, a small proportion can be detected following checks of serum prolactin level, most commonly in the general practice setting. Once identified, serum prolactin levels have to be confirmed (i.e. by excluding the presence of macroprolactin and or a falsely raised level due to the hook effect) [2]. The next investigation of choice is a dedicated pituitary MRI scan (T1 and T2 weighted scans, with or without gadolinium) [3].

Treatment goals are to resume normal gonadal function, preserve other pituitary axes and alleviate or reduce the mass-effect consequences of large adenomas on nearby structures [1]. This can be achieved by the use of dopamine agonist (DA) therapy through their inhibitory effect on the lactotroph cells. DA therapy is usually very effective and tolerated by the majority of patients [4,5]. However, a small proportion develops intolerance/side-effects or resistance to treatment [6]. This small subgroup may require other treatment modalities (e.g. surgery or radiotherapy). For the majority of patients in whom good treatment outcome (clinical, biochemical and radiological) is observed, detailed patient education on the DA side-effects and outcomes of tumour shrinkage has to take place from the first appointment.

## Case presentation

**Figure 1:**
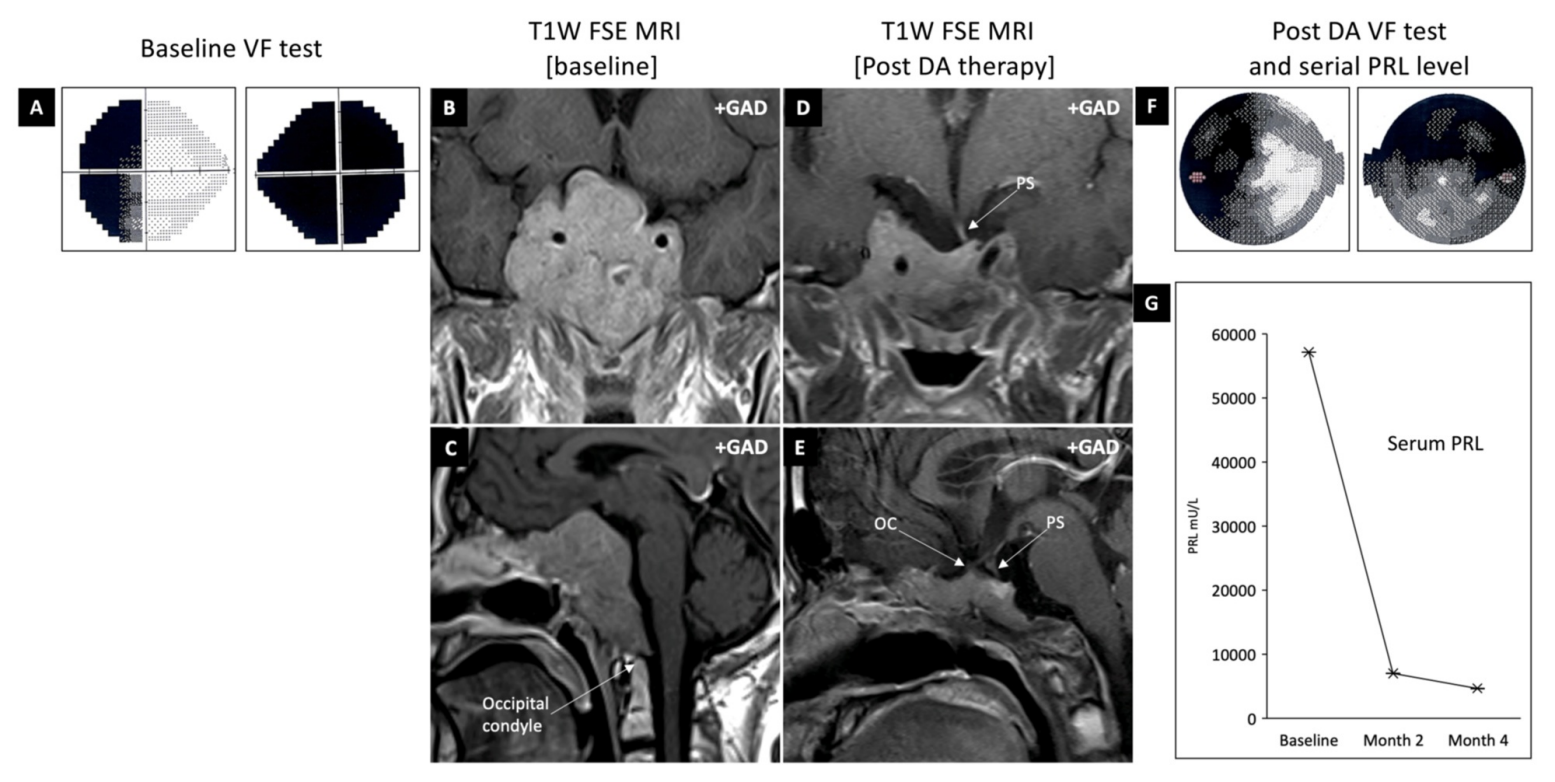
Patient clinical data Showing baseline visual fields (A) and coronal and sagittal pituitary MRI (B & C). The baseline scan shows a large skull base tumour in keeping with a pituitary macroadenoma. The tumour extends superiorly to compress the optic chiasm, inferiorly to reach the occipital condyles, and invades the cavernous sinus bilaterally (Knosp grade 4). Treatment with DA therapy results in tumour shrinkage (D & E), improvement in visual fields (F) and reduction in serum prolactin level (G). Abbreviations: DA, dopamine agonists; FSE, fast spin echo; GAD, gadolinium; MRI, magnetic resonance imaging; OC, optic chiasm; PS, pituitary stalk

**Figure 2:**
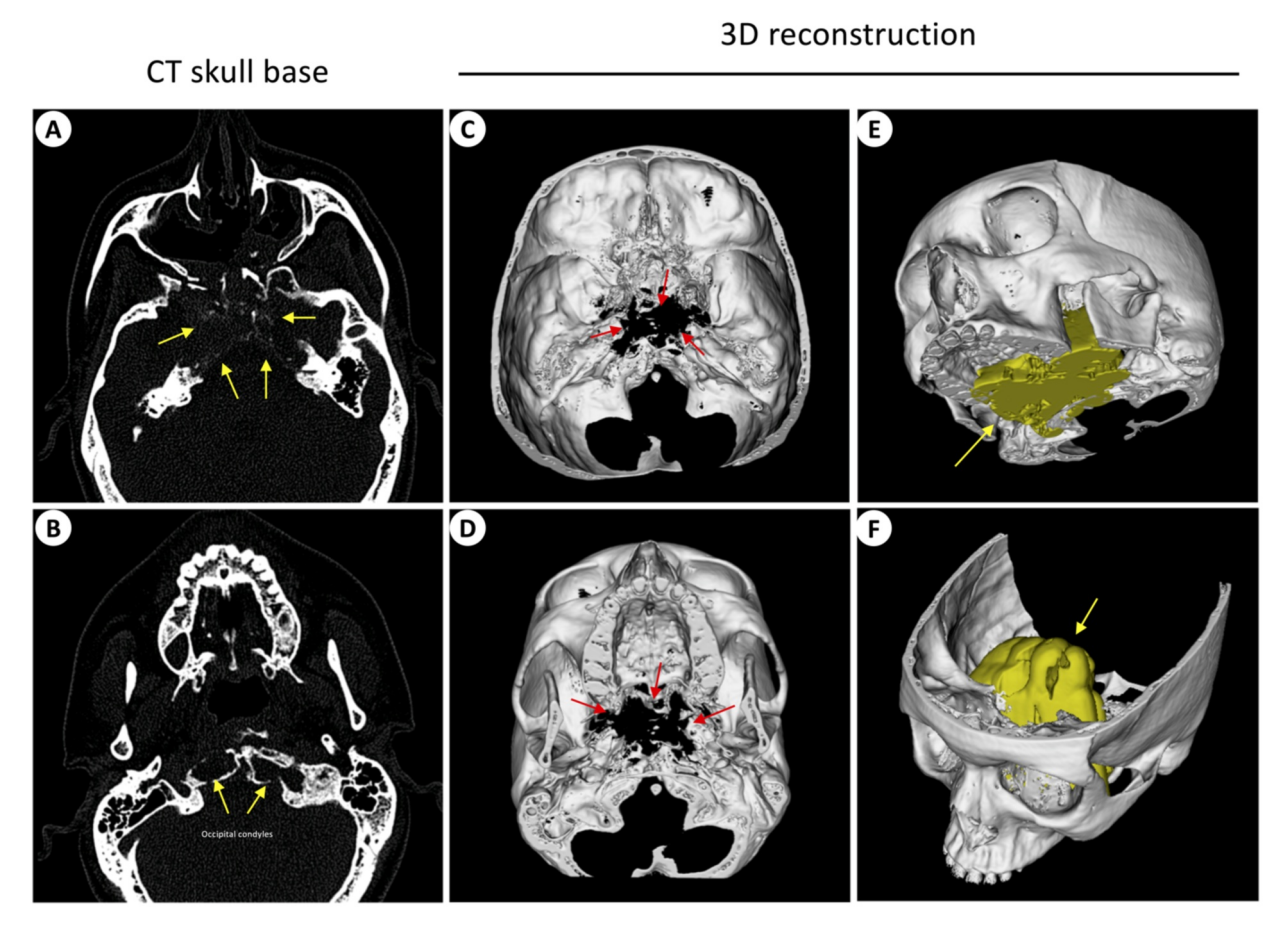
Skull CT Panels A and B show a large bony defect in the middle skull base (yellow arrows) extending inferiorly to the anterior aspect of the foramen magnum. Computerized reconstruction of the skull (C & D) shows the extent of the defect (red arrows), and when co-registered with the 3D-rendered tumour from the contemporaneous MRI scan (E & F, yellow arrows) confirms a near-total replacement of the middle skull base with large tumour tissue

A 20-year-old man presented to our yearly outreach endocrine clinic with a one-year history of decreased libido and visual deterioration. Clinical examination showed a dense bi-temporal hemianopia (worse on the right side) which was verified by a formal perimetry assessment (Figure 1A). He had no clinical features of endocrinopathy. Initial investigations confirmed central hypogonadism, elevated serum prolactin of 57,136 mU/l (reference range = 85-304) and normal pituitary functions otherwise (Table 1). Baseline brain MRI showed a large sellar lesion compressing the optic chiasm in keeping with a pituitary macroadenoma (Figure 1B, C). The tumour extended inferiorly to reach the occipital condyles and laterally to fully invade the cavernous sinuses. A diagnosis of large invasive macroprolactinoma was made, and treatment with DA therapy (titrated dose of cabergoline) resulted in excellent tumour shrinkage within four months (Figure 1D, E). This was accompanied by a significant improvement in vision and gonadal function and a >90% reduction in serum prolactin (Figure 1F, G). CT of the skull base showed near-total invasion of the middle skull base by tumour (Figure 2). Stringent clinical, biochemical and radiological follow-up is planned. He was referred to be assessed by the clinical geneticists.

**Table 1:** Baseline pituitary profile A 9 am blood test shows significant hyperprolactinaemia with central hypogonadism. He was otherwise eupituitary. Abbreviations: FSH, follicular-stimulating hormone; FT3, free T3; FT4, free T4; GH, growth hormone; IGF-1, insulin-like growth factor-1; LH, luteinizing hormone; PRL, prolactin; TSH, thyroid-stimulating hormone

9 am test	Value	Reference range
Prolactin	55,136	85-304 mU/l
TSH	5.0	0.34-5.6 mU/ml
FT4	1.0	0.6-1.12 ng/dl
FT3	3.1	2.5-3.9 ng/dl
FSH	0.81	1.27-19.26 mU/ml
LH	2.91	1.1-7.0 mU/ml
Testosterone	<2	13-29 nmol/l
GH	0.41	0-10 ng/ml
IGF-1	NA	11-31 nmol/l
Cortisol	304	>170 nmol/l

## Discussion

Our case represents one of the common scenarios in which a patient presents with visual deterioration and subsequently is discovered to have a pituitary macroadenoma. The very high level of prolactin confirms a diagnosis of macroprolactinoma and therefore the first-line treatment is DA therapy. Like many cases of macroprolactinoma, our patient responded well to treatment with excellent clinical, radiological and biochemical response (Figure 1). He showed good engagement and adherence to treatment from the first clinic visit. It was, therefore, reasonable to approach the topic of telemedicine and follow-up virtually to our patient with the knowledge that returning to face-to-face review is possible if needed.

**Figure 3:**
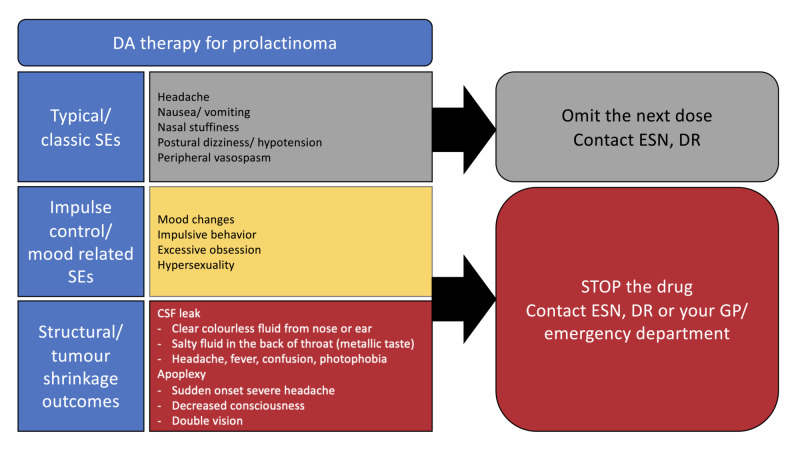
Use of DA therapy in prolactinoma, patient advice leaflet Abbreviations: CSF, cerebrospinal fluid; DA, dopamine agonists; DR, doctor; ESN, endocrine specialist nurse; GP, general practitioner; SEs, side effects

The first clinic visit, which was conducted face-to-face, involved taking a history and performing a clinical examination, followed by discussing the disease incidence, pathophysiology, genetic predisposition, investigations, treatment options, frequency of follow up visits, and prognosis. DA side-effects and treatment outcomes, in particular consequences of rapid tumour shrinkage (e.g. CSF leak or apoplexy), were stressed upon due to the nature of his tumour (large macroadenoma invading the skull base) [7]. 3D reconstruction of the tumour and the skull-based defect helped in explaining the nature of this patient's tumour to him and aided his understanding of the possible consequences of tumour shrinkage (Figure 2). A bespoke patient leaflet was provided and all possible case scenarios of use of DA therapy in this context were explained in detail (Figure 3).

 

Application of teleconsultation in this case

All of his subsequent clinic consultations were performed virtually using telemedicine methods (teleconsultation using telephone and video communications, live chat using secure texting and asynchronous written advice services). He was particularly amenable to this approach because he lived in a distant rural area with poor access to specialised clinics.

The type of technology used for each consultation was tested ahead of the appointment to minimise technical failures. Video consultations were particularly helpful in allowing limited clinical examination. It also helped in facilitating complex discussions such as symptoms of hypogonadism. Real-time texting also allowed detailed data acquisition while using a simple way of communication. Written consultation (by email and by using bespoke mini questionnaires) is also time-efficient.

Proposed telemedicine approach in prolactinomas

**Figure 4:**
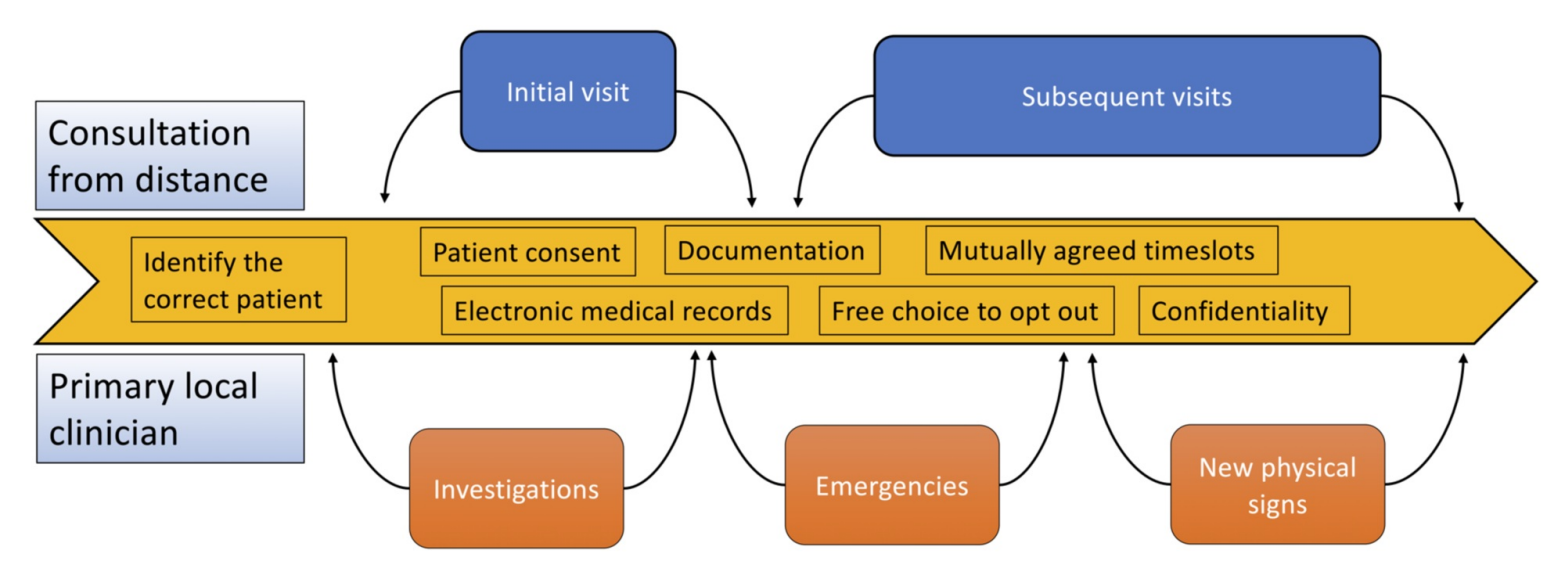
Tele-consultation ecosystem It is crucial to ensure the correct patient is selected for teleconsultation, appropriate consenting performed, and regular documentation maintained in electronic patient records. Confidentiality is paramount, particularly when considering the transfer of data between institutions.

**Figure 5:**
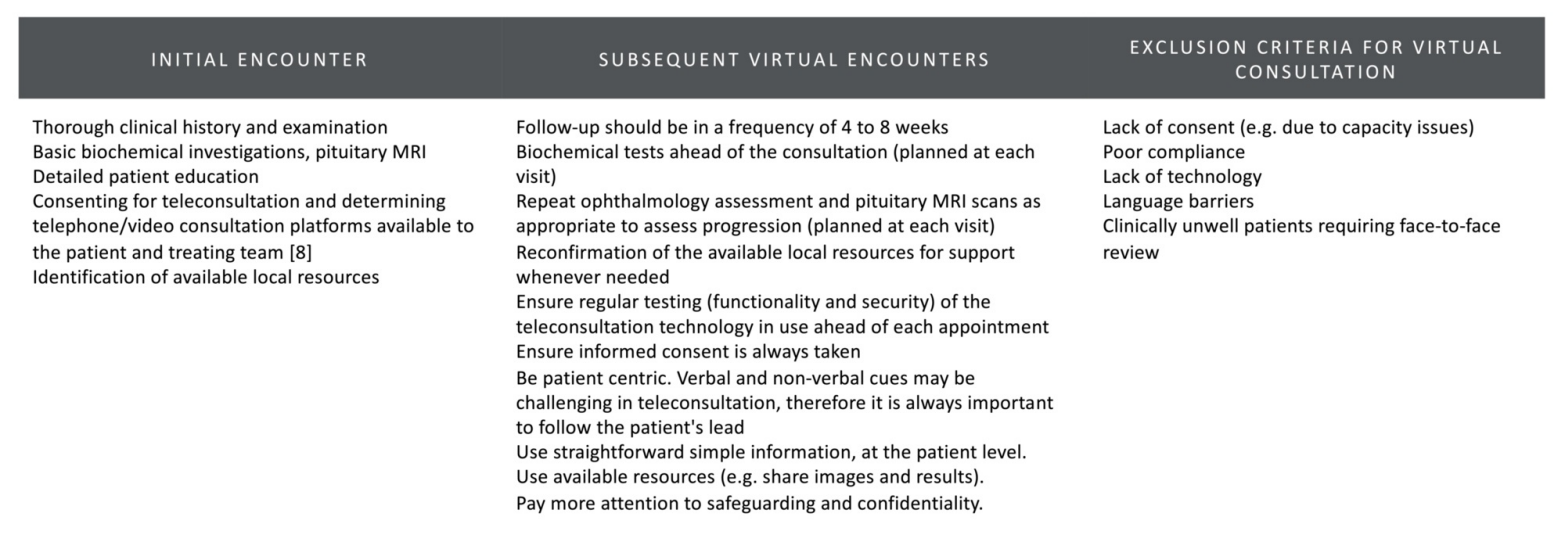
Summary guidance for virtual consultation in patients with prolactinoma

A reasonable number of patients with prolactinoma can be managed from a distance. This method has to be applied to selected patients, regularly reviewed and with guarantees of a robust availability of local resources (Figures 4 and 5).

 

Standard local setup

This should constitute a named local clinician for a face-to-face review whenever needed [8,9]. A dedicated MRI scanner (preferably the same scanner) should be used for follow-up. Also, the same laboratory should be used for each repeat biochemical measurement to avoid assay variability [10]. The patient should also have a dedicated ophthalmology centre for routine and/or urgent assessments.

## Conclusions

In conclusion, we reported a case of a macroprolactinoma in a young man which was managed using telemedicine methodology (teleconsultation) with favourable consequences. We propose the use of this approach for selected patients, with emphasis on patient empowerment, confidentiality and the swift reversal the face-to-face clinical reviews whenever needed.
